# Comparison of ultrasound with computed tomography and whole‐body diffusion‐weighted MRI in prediction of surgical outcome using ESMO‐ESGO criteria in patients with tubo‐ovarian carcinoma: prospective ISAAC study

**DOI:** 10.1002/uog.70109

**Published:** 2025-11-04

**Authors:** D. Fischerova, P. Pinto, J. L. Alcázar Zambrano, J. Vara‐Garcia, V. Chiappa, F. Raspagliesi, D. Franchi, A. V. Urbinati, A. C. Testa, J. Jarkovsky, D. Cibula, F. Moro, Collaborators, F. Frühauf, J. Slama, R. Kocian, S. Alessi, G. Avesani, K. Benesova, A. Burgetova, G. Calareso, L. Lambert, M. Masek, A. Fagotti, C. Panico, P. Pricolo, R. Vigorito

**Affiliations:** ^1^ Gynecologic Oncology Centre, Department of Gynecology, Obstetrics and Neonatology, First Faculty of Medicine Charles University and General University Hospital in Prague Prague Czech Republic; ^2^ Department of Gynecology Portuguese Institute of Oncology of Lisbon Francisco Gentil Lisbon Portugal; ^3^ First Faculty of Medicine Charles University and General University Hospital in Prague Prague Czech Republic; ^4^ QuironSalud Hospital Málaga Spain; ^5^ Department of Obstetrics and Gynecology Clínica Universidad de Navarra Pamplona Spain; ^6^ Department of Gynecologic Oncology Fondazione IRCCS Istituto Nazionale dei Tumori Milan Italy; ^7^ Preventive Gynecology Unit, Division of Gynecology European Institute of Oncology IRCCS Milan Italy; ^8^ Dipartimento Universitario Scienze della Vita e Sanità Pubblica, Università Cattolica del Sacro Cuore Rome Italy; ^9^ Dipartimento Scienze della Salute della Donna del Bambino e di Sanità Pubblica, Fondazione Policlinico Universitario Agostino Gemelli, IRCCS Rome Italy; ^10^ Institute of Biostatistics and Analyses, Faculty of Medicine Masaryk University Brno Czech Republic; ^11^ UniCamillus‐International Medical University Rome Italy

**Keywords:** computed tomography, genital neoplasm, imaging, magnetic resonance imaging, ovarian cancer, staging, surgical outcome, ultrasonography, ultrasound

## Abstract

**Objective:**

To test the non‐inferiority of extended abdominopelvic ultrasound examination compared with contrast‐enhanced computed tomography (CT) and whole‐body diffusion‐weighted magnetic resonance imaging (WB‐DWI/MRI) in discriminating preoperatively between resectable and non‐resectable disease based on the European Society for Medical Oncology (ESMO) and European Society of Gynecological Oncology (ESGO)‐defined criteria in patients with tubo‐ovarian carcinoma.

**Methods:**

The Imaging Study on Advanced ovArian Cancer was a prospective multicenter observational study conducted in five European gynecological oncology centers. All centers had ESGO accreditation to perform advanced ovarian cancer surgery, and ultrasound examinations were performed by a European Federation of Societies for Ultrasound in Medicine and Biology level‐III examiner in a standardized manner. Included in the analysis were patients enrolled between 2020 and 2022 with suspected or histologically proven primary tubo‐ovarian (including peritoneal) carcinoma who, for the purposes of the study, underwent ultrasound and CT imaging, as well as WB‐DWI/MRI if available, prior to surgery. The index tests, which included the preoperative imaging modalities as well as intraoperative exploration at the start of surgery, supplemented by biopsy or follow‐up imaging for extra‐abdominal locations, evaluated the presence of disease at eight anatomical sites that, if infiltrated, would indicate non‐resectability of the tumor according to the ESMO‐ESGO criteria. Surgical outcome, described by the surgeons at the end of the procedure, was used as the reference standard and non‐resectability was defined as the presence of residual disease > 1 cm or when debulking surgery was not feasible. The area under the receiver‐operating‐characteristics curve (AUC) and F_1_ score were used to assess the performance of the preoperative imaging methods and surgical exploration in discriminating between patients with resectable and those with non‐resectable disease, based on the ESMO‐ESGO criteria. We also calculated the percentage agreement between imaging findings and surgical exploration findings at the start of surgery, supplemented when applicable by biopsy or follow‐up imaging for extra‐abdominal locations, regarding the presence of tumor infiltration at each of the eight anatomical sites associated with non‐resectability.

**Results:**

Of 279 patients enrolled during the study period, 242 were included in the final analysis. In the subgroup of 167 patients who underwent surgery and had been examined by all three imaging methods, the AUC of the three imaging modalities and surgical exploration for discriminating between resectable and non‐resectable disease based on the ESMO‐ESGO criteria was 0.835 (95% CI, 0.756–0.915) for ultrasound, for CT it was 0.754 (95% CI, 0.664–0.843), for WB‐DWI/MRI it was 0.720 (95% CI, 0.626–0.814) and for surgical exploration it was 0.952 (95% CI, 0.915–0.988). Ultrasound was not inferior to CT or WB‐DWI/MRI, based on the AUC and F_1_ score, in discriminating between patients with resectable and those with non‐resectable tubo‐ovarian carcinoma. At surgical exploration, at least one non‐resectability criterion was present in 32.2% cases. The criteria observed most frequently at surgical exploration were small‐bowel involvement (23.6% of cases), diffuse deep infiltration of the root of the small‐bowel mesentery (18.2% of cases) and hepatic hilum involvement (5.4% of cases). The percentage agreement between ultrasound and surgical exploration in assessing the presence of disease in at least one of the eight anatomical sites that, if infiltrated, would indicate non‐resectability of tumor, was 83.9%, surpassing the percentage agreement with surgical exploration of both CT (77.7%) and WB‐DWI/MRI (75.8%).

**Conclusion:**

When performed by an experienced examiner, ultrasound is not inferior to either CT or WB‐DWI/MRI in discriminating between resectable and non‐resectable disease in patients with tubo‐ovarian carcinoma, based on evaluation of the presence of the disease in at least one of eight anatomical sites that, if infiltrated, would indicate non‐resectability of the tumor. © 2025 The Author(s). *Ultrasound in Obstetrics & Gynecology* published by John Wiley & Sons Ltd on behalf of International Society of Ultrasound in Obstetrics and Gynecology.

## INTRODUCTION

Ovarian cancer has the second‐highest mortality rate among gynecological cancers^1^. More than 90% of malignant ovarian tumors are designated tubo‐ovarian carcinoma (also referred to as ‘epithelial ovarian cancer’). The most common and most lethal tubo‐ovarian carcinoma is high‐grade serous carcinoma. When discussing such carcinomas, the primary site (i.e. ovary, Fallopian tube or peritoneum) should be designated, when possible. When it is not feasible to clearly delineate the primary site, these tumors can be reported as tubo‐ovarian carcinoma^2^, ^3^. Approximately 80% of these tumors are diagnosed at an advanced stage, when intraperitoneal dissemination has occurred^4^. Surgical outcome, defined as the amount of residual tumor at the end of surgery, heavily impacts patient prognosis^5^, ^6^.

**Figure 1:**
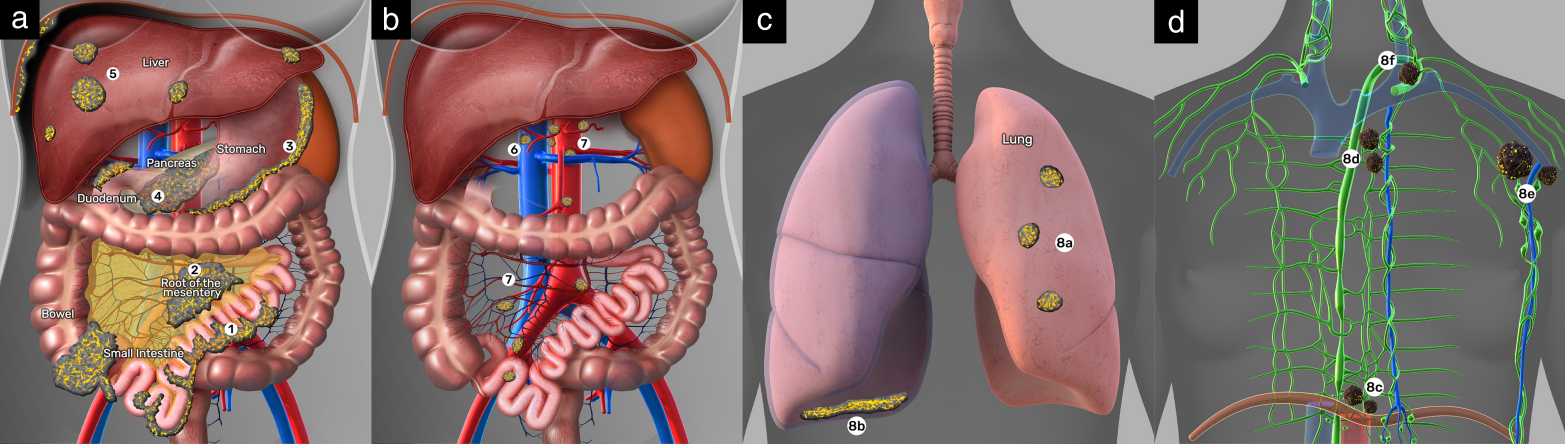
Anatomical sites for the European Society for Medical Oncology (ESMO) and European Society of Gynaecological Oncology (ESGO) criteria for non‐resectability of tubo‐ovarian carcinoma^19^: (a) small intestine (1), small‐bowel mesentery (2), stomach/duodenum (3), pancreas (4) and liver (5); (b) hepatic hilum and celiac trunk (6) and abdominal lymph nodes (7); and (c,d) extra‐abdominal sites (8). Examples of extra‐abdominal involvement: lung metastases (8a), parietal pleura (8b) and superior diaphragmatic (8c), parasternal (8d), axillary (8e) and supraclavicular (8f) lymph nodes. For detailed description of criteria, see Table 1.

It is important preoperatively, rather than intraoperatively, to be able to: (1) correctly classify primary tubo‐ovarian carcinoma in order to avoid operating on metastases from other primary tumors (secondary cancers) that are mimicking tubo‐ovarian carcinoma^7^; (2) discriminate between resectable and non‐resectable disease (non‐resectable tumor is defined as residual tumor > 1 cm after surgery^8^); (3) obtain biopsy samples for diagnosis and molecular testing in advanced disease (e.g. in patients with suspected secondary cancer or in those unsuitable for surgery owing to dissemination or poor patient condition^9^). In recently published European and international joint consensus statements, ultrasound is specified as the method of choice in the diagnosis of tubo‐ovarian carcinoma^10^, ^11^ and in image‐guided core‐needle biopsy^12^. It can also be an effective alternative to computed tomography (CT), whole‐body diffusion‐weighted magnetic resonance imaging (WB‐DWI/MRI) or positron‐emission tomography in combination with CT for assessing the extent of tumor spread and discriminating between patients with resectable and those with non‐resectable disease^11^, ^13^. To discriminate preoperatively between resectable and non‐resectable tumors, the most frequently used predictive models are the Peritoneal Cancer Index (PCI) and the Predictive Index Value (PIV), also known as the Fagotti score^8^, ^14^. Different imaging modalities, including ultrasound, have been studied to assess the parameters evaluated in PCI or PIV models^8^, ^15^, ^16^, ^17^. However, there is still a need for a simpler method with, ideally, better ability to discriminate preoperatively between resectable and non‐resectable tumors. To this end, easy‐to‐use criteria for non‐resectability of tubo‐ovarian carcinoma were first introduced in the 2017 European Society of Gynaecological Oncology (ESGO) guidelines for ovarian cancer surgery^18^ and were later adopted in the 2019 joint consensus conference recommendations of the European Society for Medical Oncology (ESMO) and ESGO, to guide surgeons in evaluating resectability^19^. Because intraoperative exploration to determine resectability is an invasive approach, it is preferable to apply the ESMO‐ESGO criteria preoperatively using imaging, thereby avoiding unnecessary surgery. In 2022, a prospective pilot study by Fischerova *et al*.^20^ demonstrated comparable performance of ultrasound, WB‐DWI/MRI and CT in discriminating preoperatively between resectable and non‐resectable disease in patients with advanced tubo‐ovarian carcinoma, using the ESMO‐ESGO intra‐abdominal non‐resectability criteria (Figure 1, Table 1, Criteria 1–7).

The objective of the present study was to test the non‐inferiority of extended abdominopelvic ultrasound examination compared with CT and WB‐DWI/MRI in discriminating preoperatively between resectable and non‐resectable disease based on the ESMO‐ESGO criteria in patients with tubo‐ovarian carcinoma.

## METHODS

### Study design

This study reports the primary endpoint of the Imaging Study on Advanced ovArian Cancer (ISAAC) study, which was a prospective, multicenter, observational study conducted in five gynecological oncology centers in three countries: the Czech Republic (General University Hospital in Prague), Italy (National Cancer Institute, Milan; European Institute of Oncology, Milan; and Fondazione Policlinico Universitario A. Gemelli, IRCCS, Rome) and Spain (Clinica Universidad de Navarra, Pamplona) (Clinicaltrials.gov: NCT03808792). The study was performed in accordance with the ISAAC protocol, current guidelines of good clinical practice^21^, the seventh revision of the Declaration of Helsinki and applicable regulatory and country‐specific requirements. The protocol was approved by the institutional review board (29/16, 04.06.2018) at the leading institution (General University Hospital in Prague) and subsequently approved by the institutional review boards of all participating institutions. Any deviations from the original protocol were included in amendments. Once a patient had provided informed consent, all three index tests (ultrasound, CT and, if available, WB‐DWI/MRI) were scheduled, and basic demographic data, including age, weight, height and body mass index, as well as menopausal status, were collected.

### Setting

All participating centers were recruited by invitation, under supervision of the independent ISAAC steering committee (Appendix S1) and study management group set up by the chief principal investigator. Selection of the participating centers was based on the ultrasound, radiological and surgical expertise of those applying, and on their agreeing with the protocol and meeting the personal and technical requirements of the evaluated methods. Recruitment in each center started only after ethics approval of that center's ethics committee had been granted.

All ultrasound examiners were required to have expertise corresponding to European Federation of Societies for Ultrasound in Medicine and Biology (EFSUMB) level‐II or level‐III^22^ and to have performed more than 100 extended abdominopelvic ultrasound examinations for staging tubo‐ovarian carcinoma within the past 5 years. The expertise of the radiologists was defined by a minimum of 5 years' practice using CT and/or MRI with a specific interest in gynecological oncology staging, including ovarian carcinoma staging. Minimum requirements regarding the surgical quality of the participating institutions were based on the quality indicators for advanced ovarian cancer surgery defined by ESGO in 2016^23^ and updated in 2020^24^. The minimum requirements were not affected by the 2020 update. The required minimum number of cytoreductive surgeries performed per center was 20 annually, with a complete resection rate of > 50% of all patients with advanced ovarian cancer (International Federation of Gynecology and Obstetrics (FIGO)^4^ Stage III–IV). The complete resection rate indicated the number of surgical procedures without residual macroscopic lesions visible at the end of surgery, relative to the total number of cytoreductive surgeries performed.

### Patients

Eligible patients were recruited consecutively between 2020 and 2022. Inclusion criteria were: (1) abdominal or pelvic mass suspicious for primary ovarian, Fallopian tubal or peritoneal carcinoma on clinical assessment or histologically confirmed primary ovarian, tubal or peritoneal carcinoma after first‐line neoadjuvant chemotherapy; (2) fitness for surgery (diagnostic laparoscopy, primary or interval debulking surgery), which was planned within 4 weeks of imaging; (3) age between 18 and 80 years; (4) Eastern Cooperative Oncology Group performance status Grade ≤ 2 (Grade 0, fully active and able to carry out all predisease performance without restriction; Grade 1, restricted in physically strenuous activity but ambulatory and able to carry out light or sedentary/office work; Grade 2, ambulatory and capable of all selfcare but unable to carry out any work activities, ‘up and about’ > 50% of waking hours)^25^; (5) not pregnant; (6) CT not contraindicated; (7) agreement to undergo two (ultrasound and CT) or three (ultrasound, CT and WB‐DWI/MRI) index tests; and (8) provided informed signed consent.

Exclusion criteria were: (1) required index tests (ultrasound, CT) not performed; (2) no reference standard available (surgery (diagnostic laparoscopy or primary or interval debulking surgery) or imaging follow‐up documenting the response to treatment in case of suspected extra‐abdominal metastases when histology of the extra‐abdominal lesion was not available); (3) interval between any imaging index test and surgery of more than 4 weeks; (4) biopsy‐proven non‐primary ovarian, tubal or peritoneal cancer, primary non‐epithelial ovarian tumor or primary epithelial benign or borderline ovarian tumor; (5) death before index tests; (6) non‐compliance with or deviation from protocol; and (7) missing data regarding index tests and/or surgery.

### Preoperative imaging

Preoperative discrimination between resectable and non‐resectable tubo‐ovarian carcinoma was based on imaging evaluation of the presence of disease at one or more predefined anatomical sites (Figure 1), according to the ESMO‐ESGO criteria of non‐resectability specified in Table 1, ^19^. According to the ISAAC protocol, the centers were encouraged to perform all three imaging modalities preoperatively in all enrolled patients. In centers in which WB‐DWI/MRI (optional test) was not available, only the mandatory tests (ultrasound and CT) were performed. Imaging tests were scheduled according to the individual center's availability of appointments. Ultrasound examiners and radiologists performing CT or WB‐DWI/MRI were instructed about the standardized approach and assessment of ESMO‐ESGO criteria of non‐resectability. The predicted surgical outcome was classified as resectable if none of the ESMO‐ESGO criteria were met and as non‐resectable if disease was detected on imaging at any one of the predefined intra‐ or extra‐abdominal sites (Criteria 1–8).

**Table 1:** European Society for Medical Oncology (ESMO) and European Society of Gynaecological Oncology (ESGO) markers of non‐resectability^19^ of tubo‐ovarian cancinoma and corresponding findings on ultrasound examination

ESMO‐ESGO marker	Anatomical site	Ultrasound definition^39^
(1) Diffuse carcinomatosis on the small‐intestine loops when resection will cause short‐bowel syndrome	Small intestine	As a result of coalescence of peritoneal carcinomatosis nodules, a diffuse, usually hypoechogenic, perfused area of tissue is formed on the serosa of the small‐intestine loops^40^. A hyperechogenic appearance of peritoneal carcinomatosis with multiple hyperechogenic spots corresponding to the presence of psammoma bodies has been described in the less frequently occurring low‐grade serous cancer^20^, ^41^
(2) Diffuse deep infiltration of the root of the small‐bowel mesentery	Small‐bowel mesentery	The mesentery of the small bowel is identified on either side of the aorta and inferior vena cava. It appears echogenic owing to the adipose content and is particularly obvious in obese patients^20^. The mesentery on the left is seen around the branches of the superior mesenteric artery with small bowel attached to its distal end. On the right, the small‐bowel mesentery is composed of adipose tissue surrounding the branches of the superior mesenteric artery and vein. If the carcinomatosis involves the small‐bowel mesentery diffusely, a cauliflower sign may be seen, resulting from retraction of the mesentery towards the small‐bowel loops (indirect sign of ‘fixity’ or ‘hypomobility’ of bowel loops toward the mesenteric root). As a direct sign, diffuse (plaque‐like) hypoechogenic perfused tumor tissue can be visualized in the root of the mesentery
(3) Diffuse carcinomatosis and/or deep infiltration of the stomach/duodenum (only limited excision is possible)	Stomach/ duodenum	Diffuse hypoechogenic carcinomatosis or nodular carcinomatosis deeply infiltrating the muscular layer of the stomach and/or duodenum
(4) Diffuse carcinomatosis and/or deep infiltration of the head or middle part of the pancreas (tail of pancreas can be resected)	Pancreas	Diffuse hypoechogenic carcinomatosis or nodular carcinomatosis deeply infiltrating the head and/or middle part of the pancreas
(5) Non‐resectable liver metastasis (central or multisegmental)	Liver	Central or multisegmental intraparenchymal liver metastases (i.e. liver focal metastatic lesions with free hepatic tissue between lesion and capsule). Liver or splenic intraparenchymal metastases are mostly hypoechogenic
(6) Diffuse carcinomatosis and/or infiltration of the hepatic hilum and celiac trunk, including hepatic arteries and left gastric artery (celiac nodes can be resected)	Hepatic hilum and celiac trunk	Hypoechogenic confluent nodules or infiltrative mass surrounding the vessels of the hepatic hilum and celiac trunk
(7) Non‐resectable lymph‐node metastases (such as involvement of multiple visceral (mesenterial with or without celiac) lymph nodes)	Abdominal lymph nodes	Multiple hypoechogenic metastatic lymph nodes (round or spiculated shape, absence of nodal‐core sign, presence of transcapsular flow) located around mesenteric and/or celiac vessels, or any metastatic lymph nodes deeply infiltrating critical anatomical structures^26^, ^27^
(8) Non‐resectable extra‐abdominal metastasis (isolated parenchymal lung metastases, axillary, superior diaphragmatic, parasternal lymph nodes, or focal parietal pleural involvement can be resected)	Extra‐abdominal	Owing to the inherent physical limitations of ultrasound, lung intraparenchymal metastases are not detectable unless directly pleural‐based. Pleural parietal carcinomatosis, however, can be visualized on ultrasound as hypoechogenic nodular or diffuse perfused plaques with irregular outline. Infiltrated non‐resectable distant lymph‐node metastases, such as multiple (disseminated) supradiaphragmatic lymph nodes, including superior diaphragmatic (also called cardiophrenic), parasternal, axillary and supraclavicular lymph nodes, may appear on ultrasound as rounded hypoechoic lesions with transcapsular flow^26^, ^27^

For illustration of anatomical sites see Figure 1.

The results of all three index tests were available for clinical decision‐making and further management. Clinical data and test results were entered into an electronic database. For building and managing the online database, a secure web application (Research Electronic Data Capture (REDCap)) provided by Charles University, Prague, was chosen. For every examination, a separate evaluation form containing only the information of that particular examination was used and this was accessible only to the operator responsible for the examination. A table containing the ESMO‐ESGO criteria of non‐resectability was included in all online evaluation forms provided by REDCap to help the clinicians. Each operator completed the ultrasound, CT and WB‐DWI/MRI evaluation forms online in the electronic database immediately after the scan and before surgery, to ensure the prospective collection of the data. An independent employee not involved in the imaging or surgery monitored all the preoperative and surgical results for data availability and completeness. In order to eliminate potential bias, each operator was blinded to the results of the other imaging modalities (e.g. the radiologist who performed the CT scan was unaware of the results of the WB‐DWI/MRI and ultrasound). When the sonographer was also a gynecological oncologist, they did not perform both ultrasound and surgery in the same patient. Similarly, the same radiologist did not perform both CT and WB‐DWI/MRI in the same patient.

#### Ultrasound of pelvis, abdomen and extra‐abdominal lymph nodes

Patients underwent a standardized ultrasound examination using a Samsung HERA I10 (Samsung Medison, Seoul, South Korea) or Voluson E10 (GE Healthcare, Zipf, Austria) machine, equipped with a 5–9‐MHz endocavitary transducer, a 3.5–7‐MHz abdominal matrix curved transducer and a 4–13‐MHz linear array transducer. The inguinal and upper diaphragmatic (cardiophrenic) lymph nodes and the presence of pleural effusions were examined systematically^26^, ^27^ as part of the standardized extended abdominopelvic ultrasound protocol, which is illustrated in Figure S1. When ultrasound showed infiltration of inguinal and/or parietal lymph nodes, specifically those in the anterior abdominal wall or abdominal (lumbar) region, with or without thoracic involvement, the supraclavicular and axillary lymph nodes were additionally assessed. No preparation (such as fasting) was required before the extended abdominopelvic scan and no contrast agents were used. The complete scan lasted 15–20 min. The detailed, systematic approach used for pelvic and abdominal ultrasound is available on the website of the International Society of Ultrasound in Obstetrics and Gynecology: https://www.isuog.org/resource/pelvic‐imaging.html and https://www.isuog.org/resource/abdominal‐scan.html, and the methodology for inguinal and supraclavicular lymph‐node assessment is described in a Consensus Opinion^27^.

#### CT of thorax, abdomen and pelvis

CT was performed on a 128‐slice scanner (Somatom Definition Edge; Siemens Healthineers, Erlangen, Germany). The patients fasted for 4 h before the examination and drank 1 L of water or diluted iodine contrast agent 1 h before the examination to distend and enhance the bowel. At the start of the examination an iodinated contrast agent (Iomeron 350; Bracco, Milan, Italy) was administered via an intravenous cannula. The cannula was left in place during the examination and for 15 min after the examination to allow intravenous access in case of an allergic reaction. The examination was performed at end‐inspiration in the venous phase from the neck to the proximal thighs. Acquisition of data took up to 2 min, depending on the patient's height and weight. The images were reconstructed in slices of 0.75 mm thickness, with 5‐mm multiplanar reconstruction. The examination lasted about 5 min.

#### WB‐DWI/MRI

WB‐DWI/MRI was performed with parallel radiofrequency transmission and phased‐array surface coils on a 3‐Tesla MRI scanner (MAGNETOM Skyra; Siemens Healthineers, Erlangen, Germany or Ingenia Elition 3T; Philips, Best, The Netherlands) with a wide‐bore MRI system (open bore diameter, 70 cm). The patient drank 1 L of water or pineapple juice to distend the bowel 1 h before WB‐DWI/MRI. At the start of abdominal WB‐DWI/MRI, an antiperistaltic agent (scopolamine butylbromide, 20 mg intravenously; Buscopan, Boehringer Ingelheim, Ingelheim am Rhein, Germany) was administered via an intravenous cannula. The cannula was left in place during the examination but was removed immediately afterwards. WB‐DWI/MRI was performed as multistation acquisition to cover the neck, chest, upper abdomen and pelvis, with the following sequences: T2 (with and without fat saturation), DWI with background suppression and postcontrast T1‐weighted imaging. The contrast agent used was gadobutrol (Gadovist, 0.1 mmol/kg; Bayer, Leverkusen, Germany) or gadoteridol (ProHance; Bracco). The examination lasted approximately 50–60 min depending on the patient's weight and height, and their cooperation.

### Surgical exploration

Intraoperative assessment of non‐resectability based on the ESGO‐ESMO criteria was carried out using a laparoscopic or open approach at the start of the surgical procedure. The surgeon assessed the presence of disease at all locations defined by the ESGO‐ESMO non‐resectability criteria within the abdominopelvic cavity and, when indicated by preoperative imaging and if technically feasible, at extra‐abdominal sites. The predicted surgical outcome was classified as resectable if no intra‐abdominal non‐resectability criteria were met and only resectable extra‐abdominal disease was present (e.g. isolated inguinal, axillary or superior diaphragmatic (cardiophrenic) nodes, or focal pleural involvement). All other cases were classified as non‐resectable. When exploration of an extra‐abdominal site indicated by imaging was not feasible, pathological confirmation by biopsy was preferred; if biopsy was not possible, the initial imaging study that identified the lesion was used as the baseline for follow‐up assessment.

### Reference standard

The decision to treat by primary or interval debulking surgery was based on each center's guidelines and took into account medical comorbidities and disease‐related factors^28^. Standard surgical debulking was performed if cytoreduction was deemed feasible on initial surgical exploration^29^. The main reference standard for discriminating between resectable and non‐resectable tumor, for both imaging methods and surgical exploration at the start of surgery, was the surgical outcome (amount of residual disease) as described by the surgeon.

After maximum effort to remove all visible tumor, the surgeon described the surgical outcome as complete cytoreduction (R0: no macroscopic residual tumor left *in situ*), optimal cytoreduction (R1: ≤ 1 cm residual tumor), suboptimal cytoreduction (R2: > 1 cm residual tumor) or cytoreduction not feasible (as determined by initial exploration). The term ‘non‐resectable disease’ included both cases with suboptimal cytoreduction (> 1 cm residual tumor) and cases in which cytoreduction was considered non‐feasible based on intraoperative findings, and was therefore not attempted. In this situation, the surgeon was asked by the REDCap platform to specify any reasons for the suboptimal or non‐feasible surgical outcome: (a) non‐resectability (complete resection was technically not feasible owing to tumor growth or location); or (b) inoperability (patient condition could not tolerate the kind of surgery that would be necessary to resect all tumor sites). The printed or online version of the surgical evaluation form was completed by the surgeon on the same day as the surgery. If the surgeon completed the printed version, the study nurse then entered the data online.

The intraoperative findings at the start of surgery (surgical exploration), supplemented by biopsy or by follow‐up imaging for extra‐abdominal metastases, were compared with the results of the three preoperative imaging methods for the detection of disease at the eight sites included in the ESMO‐ESGO criteria of non‐resectability (Figure 1). If pathological confirmation was not feasible for extra‐abdominal metastases, evidence of carcinoma infiltration was inferred from baseline imaging (defined as partial or complete regression in platinum‐sensitive disease or progression in platinum‐refractory/‐resistant disease), which served as an alternative reference standard.

Pathology data were stored at the local pathology department using the clinical archive (slides, non‐digital or digital), and a study histopathological evaluation form was entered into the database by the leading (principal) investigator at each site. Each center's lead investigators, who were ultrasound experts, had access to their own ultrasound data that they had entered into the database, and they were also responsible for entering the corresponding histopathology data or, for extra‐abdominal metastases, the follow‐up imaging data in cases in which biopsy was not feasible.

Data cleaning was performed by a team of biostatisticians (K.B. and J.J.) and study nurses. Queries were sent to participating centers to retrieve missing information and to correct inconsistencies.

### Sample size and statistical analysis

The performance of each of the three preoperative imaging methods and of surgical exploration in discriminating between patients with resectable and those with non‐resectable disease based on the ESMO‐ESGO criteria was compared with the surgical outcome at the end of surgery and evaluated using the area under the receiver‐operating‐characteristics curve (AUC). Sensitivity, specificity, positive and negative predictive values, accuracy, F_1_ and an F_0.5_ score were also calculated. To test the non‐inferiority of ultrasound, the AUCs and F_1_ scores were compared between ultrasound and CT and between ultrasound and WB‐DWI/MRI, with *P* < 0.05 considered to indicate statistical significance^30^. *P*‐values for paired AUCs were computed using the formula of Liu *et al*.^31^ and for paired F_1_ scores they were computed using a one‐sided Z‐test for non‐inferiority. The non‐inferiority of ultrasound compared with CT and WB‐DWI/MRI was the focus of the tests.

The sample size required to achieve statistical significance at 90% power was calculated using a method developed by Obuchowski and McClish^32^ with a non‐inferiority margin of 5%. The 5% margin was chosen as the point at which meaningful clinical significance is achievable while maintaining a manageable number of patients. The estimate of the AUC for WB‐DWI/MRI and CT required for sample‐size calculation was set at 0.78, as suggested by the results of previous analyses (the ISAAC pilot study^20^). These settings resulted in a requirement for 166 patients for comparison of WB‐DWI/MRI and ultrasound and 99 patients for comparison of CT and ultrasound.

In addition, the overall, positive and negative agreement between imaging findings and surgical exploration, supplemented by biopsy or follow‐up imaging for extra‐abdominal metastases, was calculated for each anatomical site, using cross‐tabulated binary classification (presence *vs* absence of disease) according to the ESMO‐ESGO criteria of non‐resectability.

## RESULTS

In all five centers, the sonographers had EFSUMB level‐III expertise and all radiologists were dedicated to gynecological oncology imaging with > 5 years' experience. All five participating centers were accredited ESGO Centers of Excellence in advanced ovarian cancer surgery, which means that they perform at least 100 cytoreductive surgeries per year and achieve complete tumor resection in ≥ 65% of the patients undergoing surgery for ovarian cancer Stages III–IV^24^. During the course of the ISAAC study, two protocol amendments were prepared by members of the steering committee and subsequently accepted by the ethics committees of the participating centers.

### Participant characteristics

**Figure 2:**
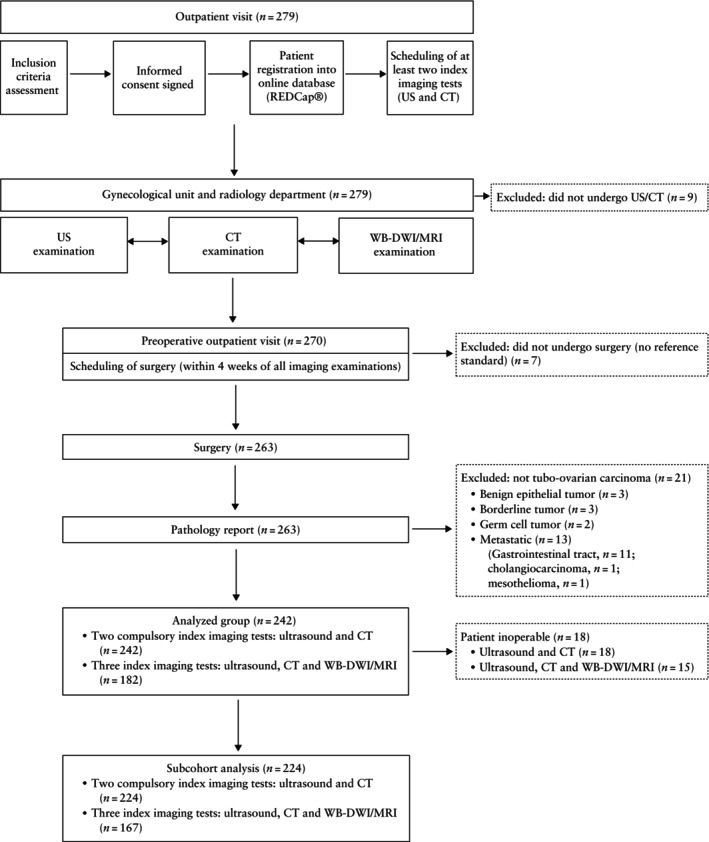
Flowchart summarizing inclusion and analysis of patients with tubo‐ovarian carcinoma. CT, contrast‐enhanced computed tomography; US, ultrasound; WB‐DWI/MRI, whole‐body diffusion‐weighted magnetic resonance imaging.

Enrolment was conducted from January 2020 until November 2022. Of 279 patients recruited, 242 were included in the final analysis (Figure 2 and Table S1). Clinical, surgical and histological characteristics of the study population are reported in Table 2. Patients had a mean age of 60.5 ± 11.1 years. The median CA 125 level was 419 U/mL. Twenty‐two (9.1%) of the 242 patients had received neoadjuvant chemotherapy before the index tests. There were 129 (53.3%) patients with ovarian carcinoma, 111 (45.9%) with tubal carcinoma and two (0.8%) with peritoneal carcinoma. In 199 (82.2%) patients, histology revealed high‐grade serous carcinoma. Regarding the abdominal surgical approach, 55 (22.7%) patients underwent only laparoscopy, 162 (66.9%) underwent only laparotomy and in 25 (10.3%) patients the approach was combined. The carcinoma was advanced (FIGO Stage III–IV) in 196 (81.0%) patients. Regarding surgical outcome, there was no residual disease at the end of surgery (R0) in 145 (59.9%) patients, optimal cytoreduction (R1) in 17 (7.0%) patients and suboptimal cytoreduction (R2) in 80 (33.1%) patients. In 62 of these 80 patients (77.5%), the inability to achieve complete or optimal cytoreduction was due to non‐resectability, while 18 (22.5%) of these patients were considered to be frail and could not tolerate extensive surgery (Table 2).

**Table 2:** Clinical, surgical and histological characteristics of study population of 242 patients with tubo‐ovarian cancer

Parameter	Value
Ultrasound examination	242 (100)
CT examination	242 (100)
WB‐DWI/MRI examination	182 (75.2)
Age (years)	60.5 ± 11.1
BMI (kg/m^2^)	25.5 ± 5.0
History of gynecological carcinoma including breast carcinoma	
Family history	62 (25.6)
Personal history	17 (7.0)
Previous neoadjuvant chemotherapy	22 (9.1)
CA 125 (U/mL)	419 (19–4105)
CEA (ng/mL)	1.8 (0.4–8.0)
Symptoms	168 (69.4)
Intraperitoneal fluid	
Overall	154 (63.6)
Abdominopelvic only (ascites)	93 (38.4)
Tense ascites*	18/93 (19.3)
Total intraperitoneal fluid volume (mL)	300 (20–5000)
*Surgery*	
Abdominal approach	
Laparoscopy only†	55 (22.7)
Laparotomy only	162 (66.9)
Combined laparoscopy and laparotomy	25 (10.3)
Type of surgery‡	
Salpingo‐oophorectomy, unilateral or bilateral	175/187 (93.6)
Hysterectomy	152/187 (81.3)
Peritonectomy	111/187 (59.4)
Rectosigmoid resection	48/187 (25.7)
Omentectomy (supracolic/infracolic)	156/187 (83.4)
Appendectomy	83/187 (44.4)
Splenectomy	17/187 (9.1)
Liver resection	4/187 (2.1)
Colon (except rectosigmoid) resection	6/187 (3.2)
Small‐bowel resection/excision	14/187 (7.5)
Diaphragmatic stripping or resection	47/187 (25.1)
Resection of stomach	3/187 (1.6)
Resection of pancreas	2/187 (1.1)
Resection of lesser omental nodules includingnodules in hepatic hilum	11/187 (5.9)
Cholecystectomy	4/187 (2.1)
Para‐aortic lymphadenectomy/sampling	63/187 (33.7)
Pelvic lymphadenectomy/sampling	60/187 (32.1)
Inguinofemoral lymphadenectomy/sampling	5/187 (2.7)
Other	16/187 (8.6)
FIGO stage	
I	33 (13.6)
II	13 (5.4)
IIIA	12 (5.0)
IIIB	20 (8.3)
IIIC	120 (49.6)
IV	44 (18.2)
Surgical outcome	
R0	145 (59.9)
R1	17 (7.0)
R2	80 (33.1)
Reason for outcome R2:	
Suboptimal cytoreduction (after maximumsurgical effort during laparotomy)	23/80 (28.8)
Cytoreduction not feasible for tumor growthand localization during laparoscopy	39/80 (48.8)
Non‐eligible for extensive surgery(inoperable)§	18/80 (22.5)
*Histology*	
Type of primary tubo‐ovarian carcinoma	
Low‐grade serous carcinoma	17 (7.0)
High‐grade serous carcinoma	199 (82.2)
Endometrioid carcinoma	9 (3.7)
Clear‐cell carcinoma	6 (2.5)
Undifferentiated carcinoma	6 (2.5)
Mucinous carcinoma	5 (2.1)
Tumor origin	
Fallopian tube	111 (45.9)
Ovary	129 (53.3)
Peritoneum	2 (0.8)

Data are given as *n* (%), mean ± SD or median (5^th^ percentile–95^th^ percentile). The following data were not available: age for seven patients, CA 125 level for seven patients and carcinoembryonic antigen (CEA) level for 84 patients.

* Tense ascites: large amount of free fluid causing symptoms of dyspnea, sleeping in vertical position (sitting) and eating only small portion size.

† Laparoscopic approach final surgical outcome: R0, *n* = 5/55; R2, *n* = 50/55 (owing to cytoreduction not feasible for tumor growth and localization (*n* = 39) or not eligible for extensive surgery (inoperable) (*n* = 11)).

‡ Type of surgery was calculated in patients who underwent laparotomy alone or laparoscopy plus laparotomy; some patients underwent more than one type of surgery.

§ Inoperability group included frail patients who could not tolerate extensive surgery (laparotomy with suboptimal surgical outcome, *n* = 7/18; diagnostic laparoscopy only, *n* = 11/18). BMI, body mass index; CT, contrast‐enhanced computed tomography; FIGO, International Federation of Gynecology and Obstetrics^4^; WB‐DWI/MRI, whole‐body diffusion‐weighted magnetic resonance imaging; R0, complete removal of all visible tumor; R1, optimal debulking with residual tumor ≤ 1 cm; R2, residual tumor > 1 cm.

### Ability of ultrasound, CT, WB‐DWI/MRI and surgical exploration to predict non‐resectability of tumor

At least one of the ESMO‐ESGO markers of non‐resectability was observed on ultrasound in 22.7% (55/242) of patients, on CT in 24.0% (58/242) of patients, on WB‐DWI/MRI in 24.2% (44/182) of patients and on surgical exploration in 32.2% (78/242) of patients (Table 3). Criterion 1 (diffuse carcinomatosis on the small‐intestine loops) and Criterion 2 (diffuse deep infiltration of the root of the small‐bowel mesentery) were the most frequently detected by surgical exploration (in 23.6% and 18.2% of cases, respectively), followed by Criterion 6 (diffuse carcinomatosis and/or infiltration of the hepatic hilum and celiac trunk, including hepatic arteries and left gastric artery) (5.4% of cases). Criteria 7 and 8 (non‐resectable lymph‐node metastases and non‐resectable extra‐abdominal metastasis) were jointly the next most frequently detected on surgical exploration (4.5% for each). In three patients, the only criterion of non‐resectability was the presence of extra‐abdominal metastasis. In one of these cases, the presence of extra‐abdominal metastases (superior diaphragmatic lymph nodes) was identified by ultrasound, WB‐DWI/MRI and CT, and was confirmed by follow‐up imaging (CT scan). In the second case, ultrasound and WB‐DWI/MRI were both false negative, with no signs of non‐resectability, while CT detected laterocervical lymph nodes of the neck as the only sign of non‐resectability (histologically confirmed). In the third case, ultrasound and WB‐DWI/MRI identified pleural carcinomatosis as the sole sign of non‐resectability, while CT was false negative; pleural carcinomatosis was later confirmed cytologically. All three patients also underwent diagnostic laparoscopy.

**Table 3:** Anatomical sites* infiltrated with cancer according to preoperative imaging and surgical exploration in 242 patients with tubo‐ovarian cancer

	Preoperative imaging			
Anatomical site involvement	US (*n* = 242)	CT (*n* = 242)	WB‐DWI/MRI (*n* = 182)	Surgical exploration (*n* = 242)†
(1) Small intestine	32 (13.2)	30 (12.4)	15 (8.2)	57 (23.6)
(2) Small‐bowel mesentery	33 (13.6)	17 (7.0)	13 (7.1)	44 (18.2)
(3) Stomach/duodenum	3 (1.2)	10 (4.1)	5 (2.7)	7 (2.9)
(4) Pancreas	2 (0.8)	6 (2.5)	1 (0.5)	4 (1.7)
(5) Liver	3 (1.2)	3 (1.2)	3 (1.6)	4 (1.7)
(6) Hepatic hilum and celiac trunk	6 (2.5)	12 (5.0)	8 (4.4)	13 (5.4)
(7) Abdominal lymph nodes	9 (3.7)	16 (6.6)	16 (8.8)	11 (4.5)
(8) Extra‐abdominal	7 (2.9)	16 (6.6)	16 (8.8)	11 (4.5)
(1) and/or (2) present	44 (18.2)	35 (14.5)	21 (11.5)	68 (28.1)
One or more markers of non‐resectability (i.e. one or more of (1)–(8)) present	55 (22.7)	58 (24.0)	44 (24.2)	78 (32.2)

Data are given as *n* (%). Not all patients had whole‐body diffusion‐weighted magnetic resonance imaging (WB‐DWI/MRI).

* Sites that, if infiltrated, are considered markers of non‐resectability according to European Society for Medical Oncology and European Society of Gynaecological Oncology criteria^19^; for detailed description of markers, see Table 1 and for illustration of anatomical sites see Figure 1.

† If surgical exploration at start of surgery was not possible (i.e. for Criterion 8, extra‐abdominal sites) then biopsy or follow‐up imaging were used as alternatives. CT, contrast‐enhanced computed tomography; US, ultrasound.

Using as the reference standard surgical outcome described by the surgeon at the end of the procedure, the performance of the three imaging modalities and surgical exploration in discriminating between patients with a resectable and those with a non‐resectable tumor using ESMO‐ESGO criteria was assessed. The 18 inoperable patients who could not tolerate extensive surgery were excluded, leaving a cohort for this analysis of 224 patients and a subcohort of 167 patients with all three imaging modalities available (Tables 4 and 5). Ultrasound proved to be non‐inferior to both CT and WB‐DWI/MRI (*P* < 0.001 based on both F_1_ score and AUC) in discriminating between resectable and non‐resectable tumor using the ESMO‐ESGO criteria (Table 5). A subgroup analysis of patients with all three imaging modalities available and without ascites (*n* = 104) found that the sensitivity of all three imaging modalities to discriminate between resectable and non‐resectable disease was reduced in the absence of ascites. However, ultrasound remained non‐inferior to both CT (*P* = 0.038 based on F_1_ score, *P* = 0.005 based on AUC) and WB‐DWI/MRI (*P* < 0.001 based on F_1_ score, *P* = 0.002 based on AUC), supporting its reliability even in patients without ascites (Table S2).

**Table 4:** Performance of ultrasound, computed tomography (CT) and surgical exploration in discriminating between resectable and non‐resectable tumors in 224 patients with tubo‐ovarian carcinoma

Procedure	AUC (95% CI)	TN (*n* (%))	FN (*n* (%))	FP (*n* (%))	TP (*n* (%))	Sens (%) (95% CI)	Spec (%) (95% CI)	PPV (%) (95% CI)	NPV (%) (95% CI)	Accuracy (%) (95% CI)	F_1_ (%) (95% CI)	F_0.5_ (%) (95% CI)
Ultrasound	0.795 (0.719–0.871)	153 (68.3)	22 (9.8)	9 (4.0)	40 (17.9)	64.5 (52.1–75.3)	94.4 (89.8–97.0)	81.6 (68.6–90.0)	87.4 (81.7–91.5)	86.2 (81.0–90.1)	72.1 (61.7–81.0)	77.5 (67.2–86.2)
CT	0.757 (0.678–0.836)	146 (65.2)	24 (10.7)	16 (7.1)	38 (17.0)	61.3 (48.8–72.4)	90.1 (84.6–93.8)	70.4 (57.2–80.9)	85.9 (79.9–90.3)	82.1 (76.6–86.6)	65.5 (54.5–74.8)	68.3 (57.2–78.3)
Surgical exploration	0.945 (0.907–0.983)	152 (67.9)	3 (1.3)	10 (4.5)	59 (26.3)	95.2 (86.7–98.3)	93.8 (89.0–96.6)	85.5 (75.3–91.9)	98.1 (94.5–99.3)	94.2 (90.3–96.6)	90.1 (84.2–95.1)	87.3 (79.4–94.0)

Inoperable patients (who could not tolerate surgery) were excluded from this analysis. Tumor non‐resectability was defined as presence of one or more markers of non‐resectability according to European Society for Medical Oncology and European Society of Gynaecological Oncology criteria^19^. Reference standard was surgical outcome (non‐resectability defined as presence of residual tumor > 1 cm or when debulking surgery was not feasible). Ultrasound was non‐inferior to CT in the prediction of tumor resectability (*P* = 0.004 based on F_1_ score and *P* = 0.006 based on area under the receiver‐operating‐characteristics curve (AUC)). F_1_, balanced F‐score (equal weight of precision and recall); F_0.5_, F‐score with higher weight of precision than recall; FN, false negative; FP, false positive; NPV, negative predictive value; PPV, positive predictive value; Sens, sensitivity; Spec, specificity; TN, true negative; TP, true positive.

**Table 5:** Performance of ultrasound, computed tomography (CT), whole‐body diffusion‐weighted magnetic resonance imaging (WB‐DWI/MRI) and surgical exploration in discriminating between resectable and non‐resectable tumors in 167 patients with tubo‐ovarian carcinoma

Procedure	AUC (95% CI)	TN (*n* (%))	FN (*n* (%))	FP (*n* (%))	TP (*n* (%))	Sens (%) (95% CI)	Spec (%) (95% CI)	PPV (%) (95% CI)	NPV (%) (95% CI)	Accuracy (%) (95% CI)	F_1_ (%) (95% CI)	F_0.5_ (%) (95% CI)
Ultrasound	0.835 (0.756–0.915)	112 (67.1)	13 (7.8)	7 (4.2)	35 (21.0)	72.9 (59.0–83.4)	94.1 (88.4–97.1)	83.3 (69.4–91.7)	89.6 (83.0–93.8)	88.0 (82.2–92.1)	77.8 (67.4–86.4)	81.0 (70.1–89.9)
CT	0.754 (0.664–0.843)	105 (62.9)	18 (10.8)	14 (8.4)	30 (18.0)	62.5 (48.4–74.8)	88.2 (81.2–92.9)	68.2 (53.4–80.0)	85.4 (78.1–90.5)	80.8 (74.2–86.1)	65.2 (52.9–75.7)	67.0 (53.6–78.6)
WB‐DWI/MRI	0.720 (0.626–0.814)	107 (64.1)	22 (13.2)	12 (7.2)	26 (15.6)	54.2 (40.3–67.4)	89.9 (83.2–94.1)	68.4 (52.5–80.9)	82.9 (75.5–88.5)	79.6 (72.9–85.0)	60.5 (47.4–71.7)	65.0 (50.8–77.1)
Surgical exploration	0.952 (0.915–0.988)	110 (65.9)	1 (0.6)	9 (5.4)	47 (28.1)	97.9 (89.1–99.6)	92.4 (86.2–96.0)	83.9 (72.2–91.3)	99.1 (95.1–99.8)	94.0 (89.3–96.7)	90.4 (83.5–95.8)	86.4 (77.5–94.0)

Inoperable patients who could not tolerate surgery were excluded from this analysis. Tumor non‐resectability was defined as presence of one or more markers of non‐resectability according to European Society for Medical Oncology and European Society of Gynaecological Oncology criteria^19^. Reference standard was surgical outcome (non‐resectability defined as presence of residual tumor > 1 cm or when debulking surgery was not feasible). Ultrasound was non‐inferior both to CT (*P* < 0.001 based on F_1_ score and area under the receiver‐operating‐characteristics curve (AUC)) and to WB‐DWI/MRI (*P* < 0.001 based on F_1_ score and AUC) in the prediction of tumor resectability. F_1_, balanced F‐score (equal weight of precision and recall); F_0.5_, F‐score with higher weight of precision than recall; FN, false negative; FP, false positive; NPV, negative predictive value; PPV, positive predictive value; Sens, sensitivity; Spec, specificity; TN, true negative; TP, true positive.

### Agreement between imaging and surgical exploration/biopsy/follow‐up

Agreement regarding infiltration of the critical anatomical sites, defined as those which, if involved, indicate tumor non‐resectability, was highest between ultrasound and surgical exploration (percentage agreement across individual criteria ranging from 84.7% to 97.9%), compared with the agreement between CT and surgical exploration and that between WB‐DWI/MRI and surgical exploration (Table S3). Notably, for Criterion 5 (non‐resectable liver metastasis), ultrasound and CT showed the same level of agreement with surgical exploration (97.9% each). The percentage agreement between ultrasound and surgical exploration in assessing infiltration by carcinoma of any one of the eight critical anatomical sites was 83.9%, outperforming both CT (77.7% percentage agreement with surgical exploration) and WB‐DWI/MRI (75.8% percentage agreement with surgical exploration).

## DISCUSSION

Our study has demonstrated that ultrasound examination performed by an experienced operator is not inferior to either CT or WB‐DWI/MRI in discriminating between resectable and non‐resectable tumors using the ESMO‐ESGO criteria in patients with tubo‐ovarian carcinoma.

The main strength of this study is its prospective design, including five oncological centers and the use of a strict, predefined protocol. All imaging operators' skill level met the minimum study requirements, and surgical quality in all centers was guaranteed by ESGO accreditation in advanced ovarian cancer surgery. All patients who met the inclusion criteria were enrolled into the study. A limitation of the study is that not all patients included in the analysis underwent all three imaging methods, with WB‐DWI/MRI performed in 75% of the patients. This was because WB‐DWI/MRI was not available in one center and some patients declined or were contraindicated to have MRI at the time of their examination. However, the required sample size (*n* = 166) was reached for a subcohort with all three imaging modalities (*n* = 167). We also found that the AUC for the performance of ultrasound and surgical exploration in the prediction of patients with resectable and those with non‐resectable disease was slightly higher in the subcohort of patients who underwent WB‐DWI/MRI than in the whole population, but this difference was not statistically significant. Another limitation is that both laparoscopic and laparotomic findings were used as the reference standard, and laparoscopy is known to be less precise in assessing intraoperative findings and intra‐abdominal parameters of non‐resectability. For ethical reasons, laparotomy was reserved for patients deemed eligible for extensive cytoreduction based on multidisciplinary assessment of clinical and imaging findings. When non‐resectable disease was suspected, diagnostic laparoscopy was performed initially, to avoid delaying chemotherapy in non‐resectable cases while preserving the chance of complete cytoreduction of potentially resectable tumors. If resectability was confirmed during diagnostic laparoscopy, the procedure was converted to laparotomy; otherwise, only a biopsy was performed.

Using the ESMO‐ESGO criteria to discriminate between patients with resectable and those with non‐resectable tubo‐ovarian carcinoma^19^, our multicenter study confirmed the promising results of our 2022 pilot study^20^, and supports the role of ultrasound as an effective alternative to both CT and WB‐DWI/MRI in assessing these patients (Table S4). It might be argued that ultrasound cannot assess extra‐abdominal spread, but studies have shown its ability to detect involvement of the pleura or scalene or axillary lymph nodes^27^, ^33^, ^34^. In our study, the presence of extra‐abdominal metastases was the sole criterion of non‐resectability in only three (1.2%) cases. Each of these was detected by at least one of the index imaging tests, but no single test identified all three: ultrasound and WB‐DWI/MRI missed one case, while CT missed another.

Many studies have evaluated the performance of imaging methods in assessing non‐resectability in patients with tubo‐ovarian carcinoma, as reported by Pinto *et al*.^8^ in a recent review. The authors found that the imaging modalities had high specificity but low sensitivity for discriminating between resectable and non‐resectable tumors. The largest prospective studies to date assessing the ability of ultrasound to discriminate between resectable and non‐resectable tubo‐ovarian carinoma were published by Testa *et al*.^35^ in 2012 and Moruzzi *et al*.^15^ in 2022 (both from the same institution). The first of these studies^35^ included 147 patients with ovarian cancer and created a scoring system based on ultrasound features to predict suboptimal cytoreduction, reporting a sensitivity of 31% and a specificity of 92%. In the second study^15^, a larger population (264 patients) was included and the two most frequent ESMO‐ESGO criteria of non‐resectability (miliary small‐bowel carcinomatosis and mesenteric retraction) were assessed on ultrasound and compared with the reference standard (laparoscopy). In comparison with our study, Moruzzi *et al*.^15^ found similar agreement between ultrasound and surgical exploration in the assessment of miliary small‐bowel carcinomatosis (86.8% *vs* our finding of 84.7%), but worse agreement in the assessment of mesenteric retraction (86.5% *vs* our finding of 91.3%). This difference could be explained by the different study design (single center *vs* our multicenter) and different reference standard (only laparoscopy *vs* our laparoscopy and/or laparotomy).

In the present study, our objective was to differentiate between resectable and non‐resectable tumors. Our findings demonstrate low sensitivity across all three imaging methods in predicting non‐resectable disease, which could result in a significant number of patients undergoing unnecessary surgery and experiencing delays in chemotherapy. However, the specificity of all methods was high, suggesting that, based on imaging, only a relatively small proportion of cases would be deprived of complete cytoreduction. It is important to emphasize that, in this clinical context, high specificity is paramount, as it is more critical to avoid depriving potentially cytoreducible patients of surgery than to subject patients to unnecessary surgery. Imaging methods provide not only information on resectability but also detailed insight into the extent of the disease, which is crucial for planning the multidisciplinary management team, comprising surgeons, anesthesiologists, nursing staff, nutritionists and physiotherapists, as well as for determining the timing and extent of debulking surgery^36^. Another implication of our study is that, since all imaging methods demonstrated similar performance, centers can select the preoperative imaging technique based on the expertise available within their team. For example, in centers with a dedicated sonographer experienced in extended abdominopelvic oncological scanning, ultrasound can be used not only for diagnosis but also for comprehensive assessment of tumor spread, including sites that are difficult to resect. In contrast, centers without such expertise should use CT or WB‐DWI/MRI to evaluate disease extent preoperatively, following an initial transvaginal ultrasound diagnosis of ovarian cancer.

Future studies should explore multimodal approaches that integrate preoperative biopsy results, radiomic features and artificial intelligence applied to imaging techniques in patients with tubo‐ovarian carcinoma. For instance, combining results from imaging methods (e.g. ultrasound, MRI or CT) with biopsy data and radiomic features extracted from tumors could enhance the preoperative selection of patients who will not benefit from extensive surgery owing to the intrinsic aggressive tumor biology^37^, ^38^. Additionally, deep‐learning models could be developed to detect automatically disease extension at preoperative imaging (ultrasound, CT or MRI) and differentiate between resectable and non‐resectable disease.

## Supporting information


**Appendix S1** Steering committee members.

**Figure S1** Schematic diagram of standardized scanning protocol for ovarian cancer.

**Table S1** Overview of patients excluded from the study, according to center.
**Table S2** Performance of ultrasound, computed tomography, whole‐body diffusion‐weighted magnetic resonance imaging and surgical exploration in discriminating between resectable and non‐resectable tubo‐ovarian carcinoma in patients without ascites who underwent all three imaging modalities (*n* = 104).
**Table S3** Overall, positive and negative agreement between each imaging modality (ultrasound, computed tomography, whole‐body diffusion‐weighted magnetic resonance imaging) and surgical exploration in assessing disease involvement of anatomical sites included in the ESMO‐ESGO non‐resectability criteria.
**Table S4** Performance of ultrasound, computed tomography, whole‐body diffusion‐weighted magnetic resonance imaging and surgical exploration in discriminating between resectable and non‐resectable tubo‐ovarian carcinoma based on ESMO‐ESGO criteria in multicenter and single‐unit study^20^.

## Data Availability

Data available on request from the authors.
