# Supplementary material for: Comparison of ultrasound with computed tomography and whole‐body diffusion‐weighted MRI in prediction of surgical outcome using ESMO‐ESGO criteria in patients with tubo‐ovarian carcinoma: prospective ISAAC study
Source: Ultrasound Obstet Gynecol. 2025 Nov 4;67(2):207–19. doi: 10.1002/uog.70109 (PMC12865518; doi:10.1002/uog.70109)
Supplement: Supplementary file 1 — Appendix S1 Steering committee members. [file UOG-67-207-s003.docx]

**Appendix S1** Steering committee members

**Daniela Fischerova**, Trial chair, gynecologic oncologist, Department of Gynecology, Obstetrics and Neonatology, First Faculty of Medicine, Charles University and General University Hospital in Prague, Czech Republic

**David Cibula**, Trial co-chair, gynecologic oncologist, Department of Gynecology, Obstetrics and Neonatology, First Faculty of Medicine, Charles University and General University Hospital in Prague, Czech Republic Charles University, Czech Republic

**Marketa Wiesnerova**, Biostatistician, Institute of Biostatistics and Analysis, Faculty of Medicine, Masaryk University, Brno, Czech Republic

**Ladislav Dusek**, Biostatistician and Director of Institute of Health Information and Statistics of the Czech Republic (ÚZIS ČR), Prague, Czech Republic (Also affiliated with Masaryk University, Brno, for academic roles)

**Martina Borcinova**, Head of Science and Research, Department of Gynecology, Obstetrics and Neonatology, First Faculty of Medicine, Charles University and General University Hospital in Prague, Czech Republic

**Ivana Nohova**, Central and Eastern European Gynecologic Oncology Group (CEEGOG) Manager, Czech Republic
