# Supplementary material for: Comparison of ultrasound with computed tomography and whole‐body diffusion‐weighted MRI in prediction of surgical outcome using ESMO‐ESGO criteria in patients with tubo‐ovarian carcinoma: prospective ISAAC study
Source: Ultrasound Obstet Gynecol. 2025 Nov 4;67(2):207–19. doi: 10.1002/uog.70109 (PMC12865518; doi:10.1002/uog.70109)
Supplement: Supplementary file 2 — Figure S1 Schematic diagram of standardized scanning protocol for ovarian cancer. [file UOG-67-207-s001.pdf]

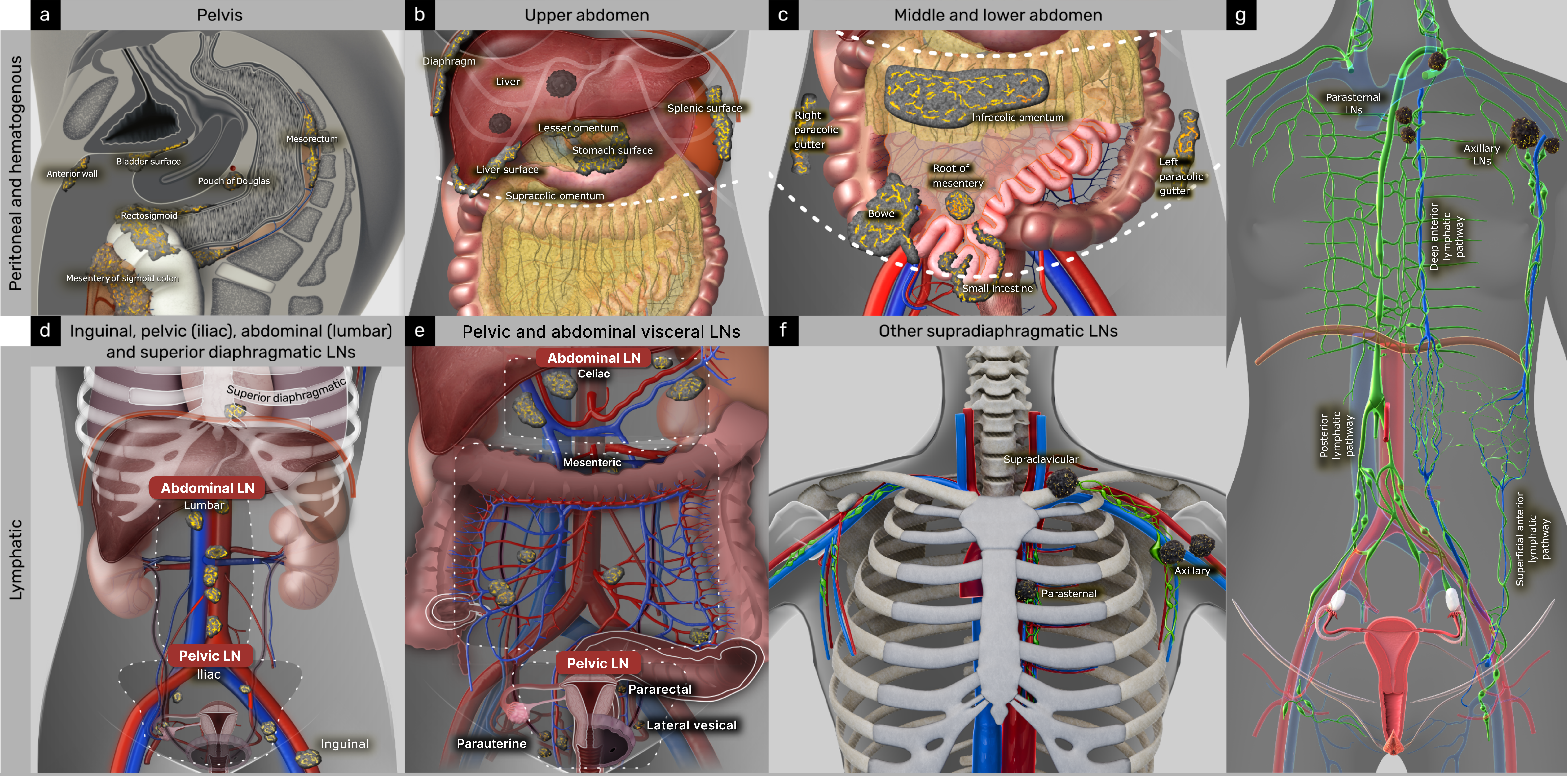

Figure S1 Schematic diagram showing how to scan advanced ovarian cancer for staging

(a-c) Peritoneal and hematogenous involvement; hematogenous metastases of the liver are depicted in panel b. (a) Pelvic involvement of parietal peritoneum (anterior wall, pouch of Douglas), visceral peritoneum (bladder and rectosigmoid surface) and mesenterial peritoneum (mesorectum, mesentery of sigmoid colon [sigmoid mesocolon]). (b) Upper abdominal involvement of parietal peritoneum (diaphragm), visceral peritoneum (surface of spleen, liver, stomach), lesser omentum and in addition hematogenous spread in liver (intraparenchymal focal metastatic lesions). (c) Middle and lower abdominal involvement of infracolic portion of greater omentum, parietal peritoneum (paracolic gutters), visceral peritoneum (bowel and small bowel serosa) and mesenterial peritoneum (root of the mesentery). **(d,e) Lymphatic involvement (mandatory ultrasound evaluation).** (d) Infiltrated inguinal, pelvic (iliac), abdominal (lumbar) and superior diaphragmatic (cardiophrenic) lymph nodes (anterior group). (e) Visceral abdominal lymph-node infiltration localized around the visceral arteries (celiac lymph nodes consisting of lymph nodes in hepatic and splenic hilum, mesenteric lymph nodes consisting of lymph nodes around the superior and inferior mesenteric vessels). The pelvic visceral lymph nodes are described during the pelvic scan together with corresponding organs. **(f) Lymphatic involvement (optional assessment).** Optional ultrasound evaluation of additional supradia phragmatic lymphatic basins (parasternal, axillary and supraclavicular lymph nodes) in cases with a higher risk of distant lymph-node dissemination, particularly in the presence of extensive inguinal and/or anterior or posterior parietal abdominal with or without superior diaphragmatic (cardiophrenic) lymph nodes. **(g) Lymphatic pathways.** Demonstration of the two main lymphatic pathways of dissemination in gynecologic cancers: the posterior lymphatic pathway and the anterior (deep and superficial) lymphatic pathways, which guide the targeted search for potential routes of lymphatic spread. LN, lymph nodes.
