# Supplementary material for: Comparison of ultrasound with computed tomography and whole‐body diffusion‐weighted MRI in prediction of surgical outcome using ESMO‐ESGO criteria in patients with tubo‐ovarian carcinoma: prospective ISAAC study
Source: Ultrasound Obstet Gynecol. 2025 Nov 4;67(2):207–19. doi: 10.1002/uog.70109 (PMC12865518; doi:10.1002/uog.70109)
Supplement: Supplementary file 3 — Table S1 Overview of patients excluded from the study, according to center. Table S2 Performance of ultrasound, computed tomography, whole‐body diffusion‐weighted magnetic resonance imaging and surgical exploration in discriminating between resectable and non‐resectable tubo‐ovarian carcinoma in patients without ascites who underwent all three imaging modalities (n = 104). Table S3 Overall, positive and negative agreement between each imaging modality (ultrasound, computed tomography, whole‐body diffusion‐weighted magnetic resonance imaging) and surgical exploration in assessing disease involvement of anatomical sites included in the ESMO‐ESGO non‐resectability criteria. Table S4 Performance of ultrasound, computed tomography, whole‐body diffusion‐weighted magnetic resonance imaging and surgical exploration in discriminating between resectable and non‐resectable tubo‐ovarian carcinoma based on ESMO‐ESGO criteria in multicenter and single‐unit study20. [file UOG-67-207-s002.docx]

**SUPPORTING INFORMATION**

**Table S1** Overview of patients excluded from the study, according to center

| **Type of center** | **No. of patients** | **Excluded** | **Included** |
| --- | --- | --- | --- |
| PCR | 136 | 16 (11.8 %) | 120 (88.2 %) |
| PSP | 32 | 3 (9.4 %) | 29 (90.6 %) |
| RIT | 28 | 10 (35.7 %) | 18 (64.3 %) |
| NIT | 60 | 3 (5.0 %) | 57 (95.0 %) |
| CIT | 23 | 5 (21.7 %) | 18 (78.3 %) |
| Total | 279 | 37 (13.3 %) | 242 (86.7 %) |

PCR=Prague, Czech Republic; PSP=Pamplona, Spain; RIT=Rome, Italy; NIT=Milan, Italy; CIT= Milan, Italy. Excluded cases were those without the ultrasound or CT imaging index test, those that did not undergo surgery (so no reference standard) and those in which there was no primary tubo-ovarian carcinoma (i.e. non-epithelial ovarian cancers, borderline tumors, metastatic cancer to the ovary) at final histology.

**Table S2** Performance of ultrasound, computed tomography, whole-body diffusion-weighted magnetic resonance imaging and surgical exploration in discriminating between resectable and non-resectable tubo-ovarian carcinoma in patients with all three imaging modalities and no ascites (*n* = 104)

|  | **AUC (95% CI)** | **TN (*n* (%))** | **FN (*n* (%))** | **FP (*n* (%))** | **TP (*n* (%))** | **Sens**  **(%) (95% CI)** | **Spec**  **(%) (95% CI)** | **PPV (%) (95% CI)** | **NPV (%) (95% CI)** | **OA (%) (95% CI)** | **F_1_ (%) (95% CI)** | **F_0.5_ (%) (95% CI)** |
| --- | --- | --- | --- | --- | --- | --- | --- | --- | --- | --- | --- | --- |
| US | 0.806 (0.664–0.948) | 84 (80.8) | 6 (5.8) | 3 (2.9) | 11 (10.6) | 64.7 (41.3-82.7) | 96.6 (90.3-98.8) | 78.6 (52.4-92.4) | 93.3 (86.2-96.9) | 91.3 (84.4-95.4) | 71.0 (48.3-87.2) | 75.3 (53.8-91.5) |
| CT | 0.771 (0.623–0.920) | 83 (79.8) | 7 (6.7) | 4 (3.8) | 10 (9.6) | 58.8 (36.0-78.4) | 95.4 (88.8-98.2) | 71.4 (45.4-88.3) | 92.2 (84.8-96.2) | 89.4 (82.0-94.0) | 64.5 (40.0-81.8) | 68.5 (44.3-87.2) |
| WB-DWI/MRI | 0.712 (0.555–0.870) | 83 (79.8) | 9 (8.7) | 4 (3.8) | 8 (7.7) | 47.1 (26.2-69.0) | 95.4 (88.8-98.2) | 66.7 (39.1-86.2) | 90.2 (82.4-94.8) | 87.5 (79.8-92.5) | 55.2 (29.6-75.0) | 61.5 (34.5-81.4) |
| EXPLOR | 0.930 (0.857–1.000) | 80 (76.9) | 1 (1.0) | 7 (6.7) | 16 (15.4) | 94.1 (73.0-99.0) | 92.0 (84.3-96.0) | 69.6 (49.1-84.4) | 98.8 (93.3-99.8) | 92.3 (85.6-96.1) | 80.0 (62.9-92.3) | 73.4 (55.0-89.7) |

In this analysis, inoperability group (patients who could not tolerate surgery) and high surgical risk group (patients who could not tolerate surgery and with non-resectable disease) were excluded. Predictor of non-resectability was presence of one or more markers of non-resectability according to ESMO-ESGO criteria^19^. The reference standard was surgical outcome (non-resectability defined as presence of residual tumor >1 cm or when debulking surgery not feasible). US is non-inferior both to CT (p = 0.038 based on F_1_ score, p = 0.005 based on AUC) and WB-DWI/MRI (p < 0.001 based on F_1_ score, p = 0.002 based on AUC). CT, computed tomography; EXPLOR, surgical exploration; F_1_, balanced F-score (equal weight of precision and recall); F_0.5_, F-score with higher weight of precision than recall; FN, false negativity; FP, false positivity; WB-DWI/MRI, whole body diffusion weighted magnetic resonance imaging; NPV, negative predictive value; OA, overall accuracy; PPV, positive predictive value; US, ultrasound; TN, true negative; TP, true positive.

**Table S3** Overall, positive and negative agreement between each imaging modality (ultrasound, computed tomography, whole-body diffusion-weighted magnetic resonance imaging) and surgical exploration* in assessing disease involvement of anatomical sites included in the ESMO–ESGO non-resectability criteria

|  |  | **Yes–yes** | **Yes–no** | **No–yes** | **No–no** | **Overall agreement (%)** | **Positive agreement (%)** | **Negative agreement (%)** |
| --- | --- | --- | --- | --- | --- | --- | --- | --- |
| **US vs. EXPLOR*** | (1) | 26 | 6 | 31 | 179 | 84.7 | 58.4 | 90.6 |
|  | (2) | 28 | 5 | 16 | 193 | 91.3 | 72.7 | 94.8 |
|  | (3) | 1 | 2 | 6 | 233 | 96.7 | 20.0 | 98.3 |
|  | (4) | 0 | 2 | 4 | 236 | 97.5 | 0.0 | 98.7 |
|  | (5) | 1 | 2 | 3 | 236 | 97.9 | 28.6 | 99.0 |
|  | (6) | 4 | 2 | 9 | 227 | 95.5 | 42.1 | 97.6 |
|  | (7) | 4 | 5 | 7 | 226 | 95.0 | 40.0 | 97.4 |
|  | (8) | 4 | 3 | 7 | 228 | 95.9 | 44.4 | 97.9 |
|  | (1–2) | 39 | 5 | 29 | 169 | 86.0 | 69.6 | 90.9 |
|  | (1–8) | 47 | 8 | 31 | 156 | 83.9 | 70.7 | 88.9 |
| **CT vs. EXPLOR*** | (1) | 20 | 10 | 37 | 175 | 80.6 | 46.0 | 88.2 |
|  | (2) | 13 | 4 | 31 | 194 | 85.5 | 42.6 | 91.7 |
|  | (3) | 0 | 10 | 7 | 225 | 93.0 | 0.0 | 96.4 |
|  | (4) | 0 | 6 | 4 | 232 | 95.9 | 0.0 | 97.9 |
|  | (5) | 1 | 2 | 3 | 236 | 97.9 | 28.6 | 99.0 |
|  | (6) | 2 | 10 | 11 | 219 | 91.3 | 16.0 | 95.4 |
|  | (7) | 4 | 12 | 7 | 219 | 92.1 | 29.6 | 95.8 |
|  | (8) | 4 | 12 | 7 | 219 | 92.1 | 29.6 | 95.8 |
|  | (1–2) | 26 | 9 | 42 | 165 | 78.9 | 50.5 | 86.6 |
|  | (1–8) | 41 | 17 | 37 | 147 | 77.7 | 60.3 | 84.5 |
| **WB-DWI/MRI vs. EXPLOR*** | (1) | 11 | 4 | 35 | 132 | 78.6 | 36.1 | 87.1 |
|  | (2) | 8 | 5 | 29 | 140 | 81.3 | 32.0 | 89.2 |
|  | (3) | 0 | 5 | 7 | 170 | 93.4 | 0.0 | 96.6 |
|  | (4) | 0 | 1 | 4 | 177 | 97.3 | 0.0 | 98.6 |
|  | (5) | 1 | 2 | 3 | 176 | 97.3 | 28.6 | 98.6 |
|  | (6) | 2 | 6 | 10 | 164 | 91.2 | 20.0 | 95.3 |
|  | (7) | 2 | 14 | 7 | 159 | 88.5 | 16.0 | 93.8 |
|  | (8) | 5 | 11 | 5 | 161 | 91.2 | 38.5 | 95.3 |
|  | (1–2) | 17 | 4 | 39 | 122 | 76.4 | 44.2 | 85.0 |
|  | (1–8) | 32 | 12 | 32 | 106 | 75.8 | 59.3 | 82.8 |

Overall, positive and negative agreement between imaging findings and surgical exploration calculated for each anatomical site using cross-tabulated binary classification (presence vs absence of disease) according to the ESMO-ESGO criteria of non-resectability. *If surgical exploration (EXPLOR) was not possible (i.e. for Criterion 8, extra-abdominal sites) then biopsy or baseline imaging were used as alternatives. Ultrasound, CT and surgical exploration available in 242 patients, WB-DWI/MRI in 182 patients. (1) Diffuse carcinomatosis on the small intestine loops when the resection will cause short bowel syndrome; (2) Diffuse deep infiltration of the root of the small bowel mesentery; (3) Diffuse carcinomatosis and/or deep infiltration of the stomach/duodenum (only limited excision is possible); (4) Diffuse carcinomatosis and/or deep infiltration of the head or the middle part of the pancreas (tail of the pancreas can be resected); (5) Non-resectable liver metastasis (central or multisegmental); (6) Diffuse carcinomatosis and/or infiltration of the hepatic hilum and celiac trunk, including hepatic arteries, left gastric artery (celiac nodes can be resected); (7) Non resectable lymph node metastases (i.e. multiple visceral (mesenterial +/- celiac) lymph nodes involvement); (8) Non-resectable extraabdominal metastasis; (1–2) = (1) and/or (2) are present; (1–8) = one or more markers of non-resectability are present. CT, contrast enhanced computed tomography; WB-DWI/MRI, whole-body diffusion weighted magnetic resonance imaging; US, ultrasound.

**Table S4** Performance of ultrasound, computed tomography, whole-body diffusion-weighted magnetic resonance imaging and surgical exploration in discriminating between resectable and non-resectable tubo-ovarian carcinoma based on ESMO-ESGO criteria in multicenter and single-unit study^20^

|  |  |  | N | AUC (95% CI) | TN (%) | FN (%) | FP (%) | TP (%) | Sensitivity  (%) | Specificity  (%) | PPV (%) | NPV (%) | OA (%) |
| --- | --- | --- | --- | --- | --- | --- | --- | --- | --- | --- | --- | --- | --- |
| Our study | US |  | 167 | 0.83 (0.756–0.915) | 112 (67.1 ) | 13 (7.8) | 7 (4.2) | 35 (21.0) | 72.9 | 94.1 | 83.3 | 89.6 | 88.0 |
| Pilot study^20^ | US |  | 67 | 0.80 (0.65-0.95) | 50 (74.6) | 6 (8.9) | 1 (1.5) | 10 (14.9) | 63.0 | 98.0 | 91.0 | 89.0 | 90.0 |
| Our study | CT |  | 167 | 0.754 (0.664–0.843) | 105 (62.9) | 18 (10.8) | 14 (8.4 ) | 30 (18.0) | 62.5 | 88.2 | 68.2 | 85.4 | 80.8 |
| Pilot study^20^ | CT |  | 67 | 0.75 (0.59-0.91) | 48 (71.6) | 7 (10.4) | 3 (4.5) | 9 (13.4) | 56.0 | 94.0 | 75.0 | 87.0 | 85.0 |
| Our study | WB-DWI/MRI |  | 167 | 0.720 (0.626–0.814) | 107 (64.1) | 22 (13.2) | 12 (7.2 ) | 26 (15.6) | 54.2 | 89.9 | 68.4 | 82.9 | 79.6 |
| Pilot study^20^ | WB-DWI/MRI |  | 67 | 0.74 (0.57-0.90) | 50 (74.6) | 8 (11.9) | 1 (1.5) | 8 (11.9) | 50.0 | 98.0 | 89.0 | 86.0 | 87.0 |
| Our study | EXPLOR |  | 167 | 0.952 (0.915–0.988) | 110 (65.9) | 1 (0.6) | 9 (5.4 ) | 47 (28.1) | 97.9 | 92.4 | 83.9 | 99.1 | 94.0 |
| Pilot study^20^ | EXPLOR |  | 67 | NA | NA | NA | NA | NA | NA | NA | NA | NA | NA |

CT, contrast enhanced computed tomography; WB-DWI/MRI, whole-body diffusion weighted magnetic resonance imaging; EXPLOR, surgical exploration; US, ultrasound. Predictor of non-resectability was presence of one or more markers of non-resectability according to ESMO-ESGO criteria (Figure 1). Reference standard for non-resectability was surgical outcome (residual tumor > 1 cm or intraoperative findings indicating that any surgical attempt to remove the cancer was not feasible). The term non-resectable disease included both cases with suboptimal cytoreduction (>1 cm residual tumor) and cases in which cytoreduction was not feasible based on intraoperative findings and was not attempted.
